# Associations between prediabetes, type 2 diabetes and incident atrial fibrillation in patients with hypertension: Results from the Swedish Primary Care Cardiovascular Database

**DOI:** 10.1016/j.ajpc.2026.101573

**Published:** 2026-03-23

**Authors:** Sara Bentzel, Tobias Andersson, Charlotta Ljungman, Jonathan SM Johansson, Per Hjerpe, Linus Schiöler, Annica Ravn-Fischer, Kristina Bengtsson Boström, Thomas Kahan, Georgios Mourtzinis

**Affiliations:** a Department of Molecular and Clinical Medicine, Institute of Medicine, Sahlgrenska Academy, University of Gothenburg, Sweden; b Department of Cardiology, Sahlgrenska University Hospital, Gothenburg, Sweden; c General Practice/Family Medicine, School of Public Health and Community Medicine, Institute of Medicine, Sahlgrenska Academy, University of Gothenburg, Gothenburg, Sweden; d Regionhälsan R&D Centre, Skaraborg Primary Care, Skövde, Sweden; e Occupational and Environmental Medicine, School of Public Health and Community Medicine, Institute of Medicine, Sahlgrenska Academy, University of Gothenburg, Gothenburg, Sweden; f Division of Cardiovascular Medicine, Department of Clinical Sciences, Danderyd Hospital, Karolinska Institutet, Stockholm, Sweden; h Department of Medicine and Emergency Care Mölndal, Sahlgrenska University Hospital, Gothenburg, Sweden

**Keywords:** Diabetes, Prediabetes, Atrial fibrillation, Hypertension, Triglyceride-glucose index, Prevention

## Abstract

**Aims:**

To study associations between prediabetes, type 2 diabetes (T2D) and insulin resistance with incident atrial fibrillation (AF) in patients with hypertension.

**Methods:**

Patients with hypertension but no AF between 2006 and 2010 were identified in the Swedish Primary Care Cardiovascular Database. Patients with type 1 diabetes or pre-existing cardiovascular disease were excluded. Patients were categorized into normoglycemia, prediabetes or T2D and followed until 2023 or incident AF. Insulin resistance was assessed using triglyceride–glucose (TyG) index and TyG–BMI index. Associations with incident AF and mortality were evaluated using multivariable models.

**Results:**

Among 15 715 patients (64 ± 11 years, 55 % women), 60 % were normoglycemic, 17 % had prediabetes and 23 % T2D. During a median follow-up of 14.7 years, AF occurred in 18 %, 21 % and 20 %, respectively. Neither prediabetes (HR 0.99, 95 % CI 0.87–1.13) nor T2D (HR 1.00, 95 % CI 0.89–1.11) was associated with incident AF compared with normoglycemia. In contrast, the TyG index demonstrated a U-shaped association with incident AF, whereas the TyG-BMI index showed a positive association. Both prediabetes and T2D were associated with increased all-cause mortality (HR 1.17, 95 % CI 1.06–1.30 and HR 1.62, 95 % CI 1.50–1.75, respectively).

**Conclusion:**

Prediabetes and T2D were not independently associated with AF in hypertensive patients. Our findings suggest a potential role for BMI and insulin resistance in AF risk, independent of glycemic category, warranting further prospective investigation.

## 1 Introduction

Atrial fibrillation (AF) is the most common form of cardiac arrhythmia and a major cause of cardiovascular morbidity and mortality [1]. Hypertension is the most significant modifiable risk factor of AF [2]. The association between type 2 diabetes (T2D) and incident AF has been investigated earlier, but findings remain inconsistent, particularly in individuals with hypertension [3,4]. Importantly, blood glucose regulation exists on a continuum with the term “prediabetes” denoting the intermediate stage between normoglycemia and T2D [5]. While prediabetes has been linked to increased cardiovascular risk [6], its relationship with incident AF among hypertensive patients remains uncertain [7,8]. Hypertension, affecting a substantial proportion of the population, frequently coexists and interacts with other cardiometabolic risk factors, including prediabetes and T2D [9]. Investigating the impact of hyperglycemic states in individuals with hypertension is therefore of particular clinical relevance.

Both prediabetes and T2D are characterized by insulin resistance [10]. Although the hyperinsulinemic-euglycemic clamp test is the gold standard for measuring insulin resistance, it is not feasible in routine care. Therefore, the triglyceride-glucose (TyG) index, assessing the product of fasting plasma glucose (FPG) and triglycerides (TG), has emerged as a surrogate marker for insulin resistance [11]. Elevated TyG index (> 8.2) has been linked to T2D development in non-obese individuals [12], while levels > 9.2 are associated with increased mortality in hypertensive patients [13]. However, the relationship between Tyg index and incident AF in a hypertensive population remains poorly studied [14].

The primary aim of this study was to examine the impact of prediabetes and T2D on the development of AF in patients with hypertension. The secondary aims were to (a) examine the relationship between TyG index and incident AF, (b) assess the associations of prediabetes, T2D and TyG index with all-cause mortality, and (c) evaluate the risk of progression to T2D among patients with prediabetes.

**Central illustration:**
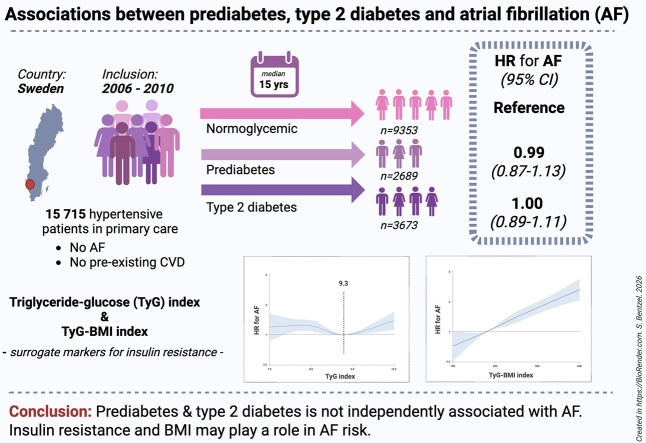

## 2 Methods

### 2.1 Data source: Swedish Primary Care Cardiovascular Database - Skaraborg

The Swedish Primary Care Cardiovascular Database (SPCCD) is a research project comprising primary health care (PHC) data on patients with hypertension, initially collected between 2001 and 2008, in two Swedish regions - Stockholm and Skaraborg [15]. SPCCD-Skaraborg (SPCCD-SKA) is an update of SPCCD, covering a wider range of patients with a longer inclusion period (2001–2014) and a longer follow-up (2001–2023), but with a smaller geographical coverage [16]. SPCCD-SKA encompasses all 20 public PHC centers in the Skaraborg county (260 000 inhabitants), representing two-thirds of all PHC centers in the area. SPCCD-SKA includes approximately 75 000 patients who visited a PHC center between 2001 and 2014 and received at least one of the following diagnosis codes according to the International Statistical Classification of Diseases - 10th Revision (ICD-10): I10-I15 hypertension; I20-I25 ischemic heart disease; I48 AF and atrial flutter (in the following here named AF); I50 heart failure; I60-I69 cerebrovascular disease; I70-I74 peripheral arterial disease; E10, E11, E14 diabetes mellitus type 1 and 2; or N18 chronic kidney disease. A purpose-built software extracted all relevant clinical data from patients' electronic medical records, including age, sex, weight, height, smoking status, systolic and diastolic blood pressure, total cholesterol, low-density lipoprotein (LDL) cholesterol, high-density lipoprotein (HDL) cholesterol, TG, creatinine, glycated hemoglobin A1c (HbA1c), FPG, and selected ICD-10 diagnosis codes registered in PHC centers’ medical records. The SPCCD-SKA is linked to national registers: the National Patient Register providing all hospital in-patient and out-patient diagnoses in Sweden [17], the Swedish Prescribed Drug Register providing data about all prescribed drugs that are dispensed in Sweden [18], the National Cause of Death Register providing all mortality data in Sweden [19] and The Longitudinal Integrated Database for Health Insurance and Labor Market Studies (LISA) providing information about the country of birth, educational level and personal disposable income for the whole population in Sweden [20].

### 2.2 Study population

This retrospective observational study with a prospective cohort design, included all patients with a registered diagnosis of hypertension (ICD-10: I10) without diagnosed AF (ICD-10: I48), and a registered FPG in SPCCD-SKA from 1 January 2006 to 31 December 2010. The studied population was followed up until 2023 for incident AF. During the study period, the recommended definition of hypertension in Sweden was an office systolic blood pressure of 140 mmHg or above and/or a diastolic blood pressure of 90 mmHg or above, measured in the supine or seated position on three separate occasions. First date with a registered FPG within the study period was considered index date. To minimize confounding, we excluded patients with pre-existing cardiovascular disease except hypertension. Exclusion criteria were defined as a registered diagnosis code of AF (I48), type 1 diabetes (E10) or cardiovascular disease (heart failure, cerebrovascular disease, ischemic heart disease and peripheral artery disease [I50, I60-I67, I69, G45, I20-I25, I70, I74]) in the PHC medical records or in the National Patient Register. We also excluded patients without a registered fasting glucose value or a blood pressure value within 730 days prior to inclusion date, or within 30 days thereafter. All included individuals were categorized in three groups: 1) normoglycemic, 2) prediabetes, or 3) T2D. Prediabetes was defined as a FPG value of 6.1–6.9 mmol/L, according to the World Health Organization’s (WHO) definition [21] and, in a secondary analysis, as a FPG value of 5.6–6.9 mmol/L, according to the American Diabetes Association’s (ADA) definition [22]. T2D was defined as either a registered diagnose code (E11-E14) in the PHC medical records or in the National Patient Register at any time before the index date and up to 30 days afterwards, or a FPG ≥ 7.0 mmol/L at index date.

### 2.3 Study variables

Data on FPG, HbA1C, total cholesterol, LDL cholesterol, HDL cholesterol, TG, creatine, blood pressure levels, weight, height, body mass index (BMI), smoking status were obtained from the PHC center’s medical records. Blood chemistry values were used if registered within two years prior to and 30 days after index date. Estimated glomerular filtration rate (eGFR, mL/min/1.73 m^2^) was calculated using the 2021 Chronic Kidney Disease Epidemiology Collaboration (CKD-EPI) equation [23]. TyG index was calculated using the following formula: Ln (fasting TG [mg/dL] × fasting glucose [mg/dL]/2) [24]. Data on comorbidity that might affect the outcome of incident AF, i.e. chronic obstructive pulmonary disease (COPD), Obstructive Sleep Apnea (OSA), gout, rheumatoid arthritis and cancer, registered at any time before the index date and up to 30 days afterwards, were extracted from the PHC center’s medical records or the National Patient Register. Educational level was obtained from the LISA Register and classified as: (a) primary and secondary education (< 12 years), (b) completed secondary education (12 years), (c) postsecondary education of less than 3 years (> 12 and < 15 years) and (d) postsecondary education of 3 years or longer (≥15 years). Data on dispensed cardiovascular medications within 180 days prior to the index date were retrieved from the Swedish Prescribed Drug Register, using anatomical therapeutic chemical (ATC)-codes. Drug classes assessed were angiotensin-converting enzyme inhibitors, angiotensin receptor blockers, thiazide (including thiazide like) diuretics, loop diuretics, mineralocorticoid receptor antagonists, beta blockers, calcium channel blockers, statins, ezetimibe, acetylsalicylic acid, P2Y12-inhibitors and oral anticoagulation (for ATC-codes; see supplementary Table S1).

### 2.4 Outcome definitions

The primary outcome of the study was incident AF, defined as the occurrence of a registered diagnose code of AF (I48). Secondary outcomes were all-cause mortality and incident type 2 diabetes, defined as a registered diagnose code of E11-E14. Data were obtained from the PHC centers’ medical records, or the National Patient Register, or the National Cause of Death Register. All patients were followed until incident AF, emigration, death or end of follow-up (31 December 2023).

### 2.5 Statistics

The studied population is presented with mean values ± standard deviations or frequences and percentages, as appropriate. Hazard ratios (HR) for the associations of prediabetes, T2D and incident AF were calculated using Cox proportional hazards models with age as the time scale and normoglycemia as the reference. All continuous variables were included as cubic restricted splines in the regression model, with knots placed at the 5th, 35th, 65th and 95th percentiles. Model 1 was unadjusted with age as the time scale. Model 2 was as Model 1 plus adjusted for baseline BMI. Model 3 was adjusted as Model 2 plus sex and baseline systolic blood pressure. Model 4 was adjusted as Model 3 plus the baseline variables; eGFR, concomitant cardiovascular medication and educational level. Because of missing data for some variables, not all patients were included in the Cox regression analyses. Data on smoking status were excluded from the analyses due to a high proportion of missing data and the inability to distinguish former smokers from current smokers. Sensitivity analyses were performed using the ADA’s definition of prediabetes (FPG 5.6–6.9 mmol/L).

To address the secondary aims of the study, we employed Cox proportional hazards models, with all models adjusted according to Model 4. The association of TyG index and incident AF was evaluated using age as the time scale, with the TyG index value corresponding to the lowest risk of AF set as the reference. Likewise, the associations of prediabetes and T2D with all-cause mortality were assessed using age as the time scale, with normoglycemia set as the reference. The association between TyG index and all-cause mortality was evaluated using the same time scale. Finally, the associations between prediabetes and T2D development were assessed with age as the time scale. For analyses of TyG index we conducted the following sensitivity analyses: excluding BMI values, using multiple imputation of missing values as well as sex-separated analyses. Further sensitivity analyses for insulin resistance were conducted using TyG-BMI index ((ln [TG (mg/dL)  ×  FPG (mg/dL)/2]  ×  BMI (kg/m^2^)) [25]. A two-tailed p-value < 0.05 was considered significant. All data management and statistical analyses were performed with SAS version 9.4 TS1M6 (SAS Institute, Cary, North Carolina, USA).

The study was approved by the Regional Ethical Review Board in Gothenburg (approval no. 577–17 and T596–18) and the Swedish Ethical Review Authority (approval no. 2019–02847 and 2021–04934). The need for individual consent was waived.

## 3 Results

**Fig. 1:**
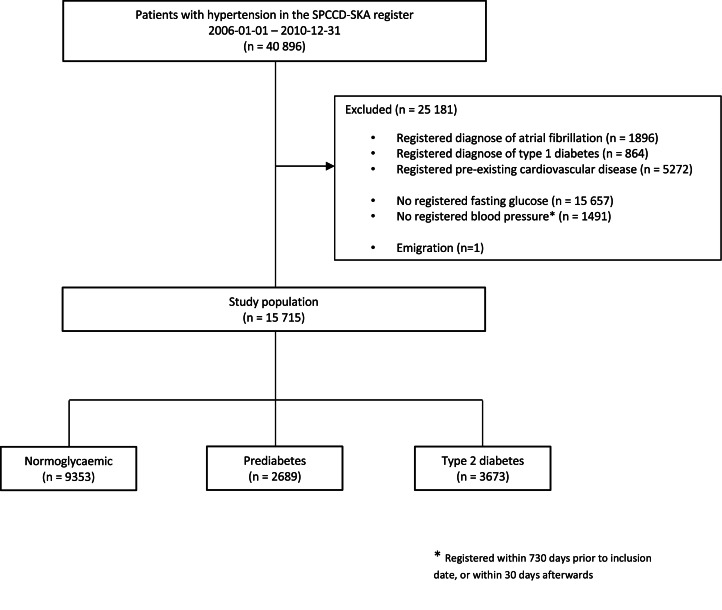
Flowchart of the study.

The study included 15 715 patients with hypertension with a mean age of 63.7 ± 11.2 years; 8693 (55.3 %) were women, flowchart of the study is presented in Fig. 1. At study start 9353 (59.5 %) were normoglycemic, 2689 (17.1 %) had prediabetes and 3673 (23.4 %) were diagnosed with T2D. Median follow-up was 14.7 years (Q1, Q3: 10.2, 16.8), yielding a total follow-up time of 203 893 person-years. The baseline characteristics and frequency of dispensed cardiovascular medications within 18 months prior to index date are presented in Table 1. During the study period, AF occurred in 1686 of normoglycemic patients, in 553 of patients with prediabetes and in 752 of patients with T2D. The overall incidence rate of AF per 1000 person-years was 14.7 (95 % confidence intervals [CI] 14.2–15.2), and for each subgroup: 13.5 (95 % CI; 12.8–14.1) in the normoglycemic population, 16.2 (95 % CI; 14.9–17.6) in patients with prediabetes, and 16.9 (95 % CI; 15.7–18.1) in patients with T2D. Numbers and incidence rates for all endpoints according to glycemic status are presented in Table 2.

SPCCD-SKA, the Swedish Primary Care Cardiovascular Database-Skaraborg

**Table 1:** Baseline characteristics and prescribed drugs within 18 months prior index date.

	All (*n* = 15 715)	Normal glycemic status (*n* = 9353)	Prediabetes (*n* = 2689)	Diabetes mellitus type 2 (*n* = 3673)	Missing values, n (%)
Male sex	7022 (44.7)	3859 (41.3)	1240 (46.1)	1923 (52.4)	0 (0)
Age at entry, years	63.7 ± 11.2	62.9 ± 11.4	64.8 ± 10.7	65.2 ± 10.9	0 (0)
BMI kg/m^2^	29.3 ± 5.3	28.2 ± 4.8	30.1 ± 5.5	30.7 ± 5.6	6117 (39)
Educational level, total years of education					157 (1)
<12	11 667 (74.2)	6690 (71.5)	2032 (75.6)	2945 (80.2)	
12	1385 (8.8)	884 (9.5)	228 (8.5)	273 (7.4)	
>12 and <15	1172 (7.5)	772 (8.3)	192 (7.1)	208 (5.7)	
≥15	1334 (7.2)	198 (5.4)	205 (7.6)	931 (10.0)	
Systolic blood pressure, mmHg	149.5 ± 19.5	149.8 ± 19.9	151.4 ± 19.5	147.5 ± 18.4	0 (0)
Diastolic blood pressure, mmHg	83.8 ± 11.3	84.8 ± 11.4	84.2 ± 11.1	81.1 ± 10.9	0 (0)
Fasting plasma glucose, mmol/L	6.3 ± 1.9	5.3 ± 0.4	6.4 ± 0.2	8.7 ± 2.7	0 (0)
HbA1c, mmol/mol	49.3 ± 15.3	37.3 ± 4.1	39.7 ± 5.2	55.4 ± 15.7	10 630 (68)
eGFR, ml/min/1.73 m^2^	87.2 ± 15.9	87.0 ± 15.6	86.8 ± 15.6	88.1 ± 16.7	604 (4)
Total cholesterol, mmol/L	5.6 ± 1.1	5.8 ± 1.1	5.6 ± 1.0	5.3 ± 1.1	1459 (9)
HDL, mmol/L	1.5 ± 0.4	1.5 ± 0.4	1.5 ± 0.4	1.3 ± 0.4	1662 (11)
LDL, mmol/L	3.4 ± 0.9	3.5 ± 0.9	3.4 ± 0.9	3.0 ± 0.9	1908 (12)
Triglyceride, mmol/L	1.6 ± 1.2	1.5 ± 0.9	1.6 ± 0.9	2.0 ± 1.9	1634 (10)
TyG index	8.8 ± 0.6	8.6 ± 0.5	8.9 ± 0.5	9.3 ± 0.7	1634 (10)
TyG-BMI index	262 ± 56	245 ± 47	268 ± 54	288 ± 59	6830 (43)
**Medication at entry**					
ACEi/ARB	5823 (37.1)	3158 (33.8)	958 (35.6)	1707 (46.5)	-
Beta blocker	5190 (33.0)	2951 (31.6)	997 (37.1)	1242 (33.8)	-
CCB (any type)	3178 (20.2)	1763 (18.8)	600 (22.3)	815 (22.2)	-
CCB (DHP type)	3066 (19.5)	1696 (18.1)	583 (21.7)	787 (21.4)	-
CCB (non-DHP type)	114 (0.7)	67 (0.7)	18 (0.7)	29 (0.8)	-
Loop diuretic	1043 (6.6)	472 (5.0)	193 (7.2)	378 (10.3)	-
MRA	978 (6.2)	529 (5.7)	204 (7.6)	245 (6.7)	-
Thiazide diuretic	4612 (29.3)	2661 (28.5)	889 (33.1)	1062 (28.9)	-
Any anti-hypertensive drug	11,856 (75.4)	6966 (74.5)	2116 (78.7)	2774 (75.5)	-
Acetylsalicylic acid	1650 (10.5)	735 (7.9)	251 (9.3)	664 (18.1)	-
OAC therapy	116 (0.7)	68 (0.7)	15 (0.6)	33 (0.9)	-
Statin	2666 (17.0)	1309 (14.0)	415 (15.4)	942 (25.6)	-
Ezetimibe	30 (0.2)	17 (0.2)	8 (0.3)	5 (0.1)	-
Insulin	351 (2.2)	0 (0.0)	1 (0.0)	350 (9.5)	-
Metformin	1229 (7.8)	3 (0.0)	8 (0.3)	1218 (33.2)	-
Sulfonylureas	647 (4.1)	0 (0.0)	2 (0.1)	645 (17.6)	-
Alpha-glucosidase inhibitor	11 (0.1)	1 (0.0)	0 (0.0)	10 (0.3)	-
Thiazolidinedione	79 (0.5)	0 (0.0)	2 (0.1)	77 (2.1)	-
DDP-4 inhibitor	4 (0.0)	0 (0.0)	0 (0.0)	4 (0.1)	-
GLP-1 receptor agonist	2 (0.0)	0 (0.0)	0 (0.0)	2 (0.1)	-
SGLT2 inhibitor	0 (0.0)	0 (0.0)	0 (0.0)	0 (0.0)	-
Repaglinide	73 (0.5)	0 (0.0)	1 (0.0)	72 (2.0)	-
**Comorbidities**					
COPD	80 (0.5)	38 (0.4)	16 (0.6)	26 (0.7)	-
OSA	213 (1.4)	127 (1.4)	33 (1.2)	53 (1.4)	-
Gout	25 (0.2)	11 (0.1)	8 (0.3)	6 (0.2)	-
Rheumatoid arthritis	74 (0.5)	51 (0.5)	9 (0.3)	14 (0.4)	-
Cancer	801 (5.1)	458 (4.9)	139 (5.2)	204 (5.6)	-

Values are presented as means ± standard deviation or frequencies (%) as appropriate.

BMI, body mass index; HbA1c, glycated hemoglobin A1c; eGFR, estimated glomerular filtration rate; HDL, high-density lipoprotein; LDL, low-density lipoprotein; TyG index, Triglyceride-Glucose Index; Tyg-BMI index, Triglyceride-BMI index; ACEi, angiotensin-converting enzyme inhibitor; ARB, angiotensin receptor blocker; CCB, calcium channel blocker; DHP, dihydropyridines; MRA, mineralocorticoid receptor antagonists; OAC, oral anticoagulation; DDP-4, dipeptidyl peptidase 4; GLP-1, glucagon-like peptide-1; SGLT2, sodium-glucose linked transporter 2; COPD, chronic obstructive pulmonary disease; OSA, obstructive sleep apnea. TyG index calculated as the Ln (fasting triglycerides [mg/dL] × fasting glucose [mg/dL]/2). TyG-BMI index calculated as the Ln ((fasting triglycerides [mg/dL] × fasting glucose [mg/dL]/2) x BMI)).

**Table 2:** Unadjusted incidence rates for atrial fibrillation, type 2 diabetes and mortality per 1000 person-years of follow-up.

	All (*n* = 15 715)	Normal glycemic status (*n* = 9353)	Prediabetes (*n* = 2689)	Diabetes mellitus type 2 (*n* = 3673)
Atrial fibrillation				
Events, *n*	2991	1686	553	752
Incidence rate (CI)	14.7 (14.2–15.2)	13.5 (12.8–14.1)	16.2 (14.9–17.6)	16.9 (15.7–18.1)
Type 2 diabetes				
Events, *n*	1675	776	899	-
Incidence rate (CI)	31.2 (30.4–32.1)	6.0 (5.6–6.5)	31.7 (29.7–33.8)	-
All-cause mortality				
Events, *n*	5420	2717	953	1750
Incidence rate (CI)	24.6 (24.0–25.3)	20.2 (19.4–21.0)	25.7 (24.1–27.4)	36.2 (34.5–37.9)

Data are numbers or incidence rate per 1000 patient years if not indicated otherwise.

Neither prediabetes nor T2D was associated with an increased risk for incident AF compared to normoglycemia in the fully adjusted Model 4 (Table 3), HR for prediabetes was 0.99 (95 % CI 0.87–1.13) and for T2D 1.00 (95 % CI 0.89–1.11). Sensitivity analyses using ADA’s definition of prediabetes yielded similar results, with HR of 1.09 (95 % CI 0.97–1.23) for prediabetes and HR 1.05 (95 % CI 0.93–1.19) for T2D. Likewise, analyses with imputed BMI values showed no association between prediabetes (HR 0.99 [95 % CI 0.89–1.09]) or T2D (HR 0.99 [95 % CI 0.90–1.08]) with incident AF.

**Table 3:** Adjusted hazard ratios for associations between prediabetes, type 2 diabetes and incident atrial fibrillation (95 % Confidence Interval).

	Model 1 (unadjusted)	Model 2	Model 3	Model 4
Normoglycemia	ref	ref	ref	ref
Prediabetes	1.11 (1.01–1.22)	1.02 (0.90–1.17)	1.00 (0.88–1.14)	0.99 (0.87–1.13)
Type 2 diabetes	1.17 (1.08–1.28)	1.04 (0.94–1.15)	1.00 (0.90–1.11)	1.00 (0.89–1.11)

Model 1: unadjusted with age as the time scale.

Model 2: as Model 1 plus adjusted for baseline Body Mass Index.

Model 3: as Model 2 plus adjusted for sex and baseline systolic blood pressure.

Model 4: as Model 3 plus adjusted for baseline estimated glomerular filtration rate, dispensed cardiovascular medication (angiotensin-converting enzyme inhibitors, angiotensin receptor blockers, thiazide diuretics, loop diuretics, mineralocorticoid receptor antagonists, beta blockers, calcium channel blockers, statins, ezetimibe, acetylsalicylic acid, P2Y12-inhibitors and oral anticoagulation) and educational level

**Fig. 2:**
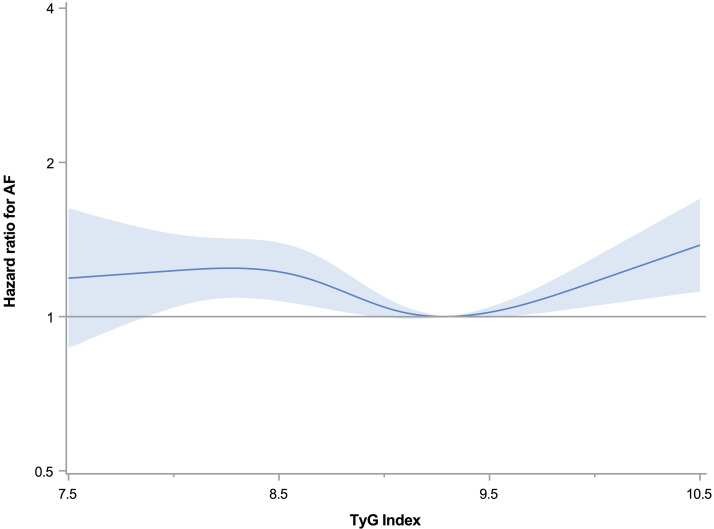
Triglyceride-glucose index association to incident atrial fibrillation.

**Fig. 3:**
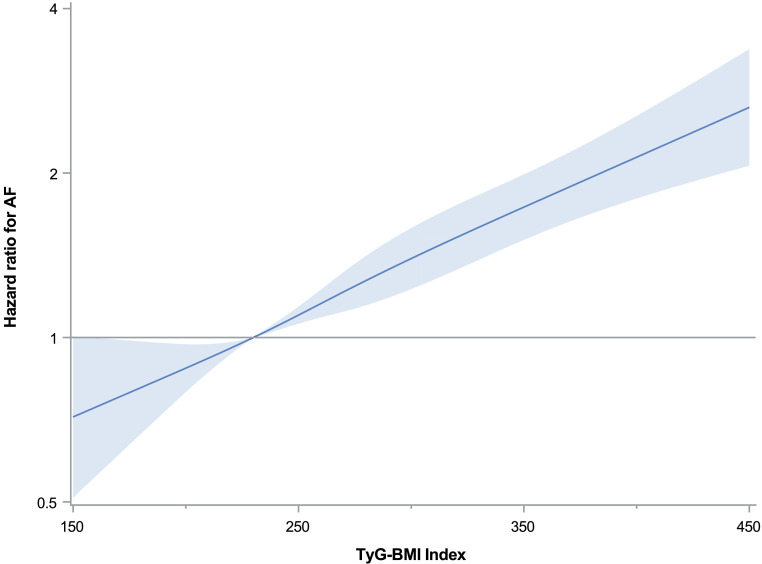
Triglyceride-glucose-BMI index association to incident atrial fibrillation.

Both low and high TyG index values were associated with a higher incidence of AF compared with the nadir at a TyG index of 9.3, (HR 1.23 [95 % CI 1.04–1.45] at TyG index 8 and HR 1.17 [95 % CI 1.05–1.31] at TyG index 10), Fig. 2. Sensitivity analyses with imputed values for BMI, stratified by different BMI values and sex-specific analyses showed the same U-shaped association, Supplementary Figure S1-S3. There was no evidence of effect modification by sex (p=0.58), nor by age or systolic blood pressure, as interaction terms were non-significant (p>0.13) and did not improve model fit as assessed by AIC. The sensitivity analysis using TyG-BMI index, instead of TyG index, showed a positive association between TyG-BMI index and incident AF, Fig. 3.

Age as the time scale, adjusted for body mass index, sex, systolic blood pressure, estimated glomerular filtration rate, concomitant cardiovascular medication and educational level. Shade areas indicate 95 % confidence interval. AF, atrial fibrillation; TyG index, Triglyceride-glucose index.

Age as the time scale, adjusted for sex, systolic blood pressure, estimated glomerular filtration rate, concomitant cardiovascular medication and educational level. Shade areas indicate 95 % confidence interval. AF, atrial fibrillation; TyG index, Triglyceride-glucose index; BMI, body mass index.

**Fig. 4:**
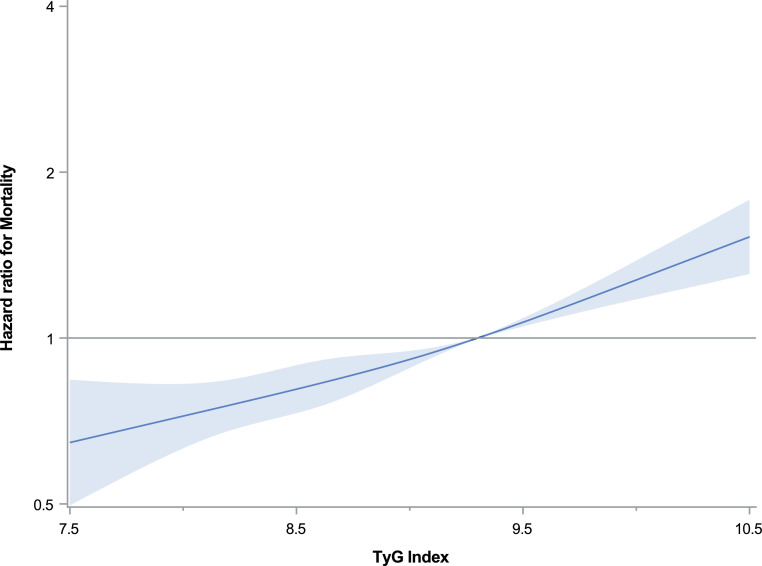
Triglyceride-glucose index association to mortality.

**Fig. 5:**
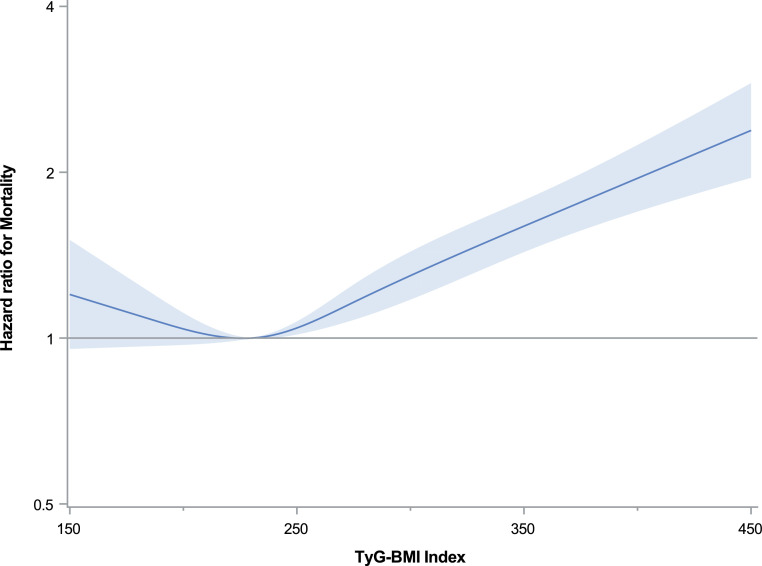
Triglyceride-glucose-BMI index association to mortality.

Both prediabetes and T2D were associated with increased mortality compared to normoglycemia in the fully adjusted Model 4 (HR 1.17 [95 % CI 1.06–1.30] for prediabetes and HR 1.62 [95 % CI 1.50–1.75] for T2D). Prediabetes was also associated with an increased risk for incident T2D, compared to normoglycemia (HR 4.47 [95 % CI 3.94–5.07]). A positive association was observed between TyG index and all-cause mortality, Fig. 4, as well as for TyG-BMI index and all-cause mortality, Fig. 5.

Age as the time scale, adjusted for body mass index, sex, systolic blood pressure, estimated glomerular filtration rate, concomitant cardiovascular medication and educational level. Shade areas indicate 95 % confidence interval. TyG index, Triglyceride-glucose index.

Age as the time scale, adjusted for sex, systolic blood pressure, estimated glomerular filtration rate, concomitant cardiovascular medication and educational level. Shade areas indicate 95 % confidence interval. TyG index, Triglyceride-glucose index; BMI, body mass index.

## 4 Discussion

There are three main findings in this large retrospective cohort study of 15 715 hypertensive individuals without previous AF or other cardiovascular disease attending PHC. First, neither prediabetes nor T2D was associated with higher risk of incident AF compared to normoglycemic status. Second, TyG index showed a U-shaped association with incident AF, while TyG-BMI index showed a positive association with incident AF. Third, we confirmed an increased mortality risk in hypertensive patients with elevated TyG index as well as with elevated TyG-BMI index.

### 4.1 Associations between prediabetes, T2D and incident AF

Our findings suggest that neither prediabetes nor T2D is independently associated with incident AF in hypertensive patients. In contrast, previous epidemiological studies and a prior meta-analysis reported up to 30 % increased risk of AF among individuals with prediabetes or diabetes [7,26,27]. However, there is considerable heterogeneity in the design among the included studies in that meta-analysis. In many cases glycemic status was not assessed as the primary exposure but rather treated as a covariate, which may have influenced the reported outcomes. In line with our findings, both a Mendelian randomization analysis and a large cohort study found no evidence supporting a causal role of type 2 diabetes or glucose metabolism in the development of AF, particularly after adjustment for confounding factors (4, 8). A summarizing comparison of conducted studies on the association between prediabetes/T2D and risk of AF is presented in Table S2.

While the unadjusted model in our study showed an increased risk for AF in both prediabetes och T2D, this association disappeared after adjustment for BMI. These findings suggest that excess body weight might be an important driver of AF in hypertensive patients, implicating an interplay of risk factors in AF development. Excess body weight may increase susceptibility to AF through several mechanisms including increased epicardial adipose tissue, left atrial enlargement and diastolic dysfunction [28]. However, hyperglycemia per se may also contribute to AF risk through mechanism such as systemic low-grade inflammation, structural remodeling and a greater burden of comorbidities [29,30]. This notwithstanding, several of these aforementioned mechanisms are modulated by both hypertension and overweight. Further studies are warranted to better understand the intricate relationship between hypertension, metabolic dysfunction, cardiovascular remodeling and risk for incident AF.

### 4.2 Association between TyG index and incident AF

In this study, both a TyG index of 8 and a TyG index of 10 were associated with increased risk of incident AF as compared to the nadir of risk observed at a TyG index value of 9.3. These findings align with the results from the ARIC study and a prospective analysis from the UK Biobank, both of which reported a similar U-shaped relationship between TyG index and incident AF [31,32]. Notably, the prevalence of hypertension in these cohorts was approximately 30 %, whereas our findings extend this non-linear relationship to a fully hypertensive population – an observation that, to our knowledge, has not been previously reported. Moreover, a recent meta-analysis reported an overall association between higher TyG index levels and AF risk, but included studies were highly heterogeneous and primarily conducted in populations with specific comorbid conditions, such as ischemic heart disease, hypertrophic cardiomyopathy and liver disease [33]. Our results expand these previous observations to a hypertensive population without pre-existing cardiovascular disease. As the TyG index reflects both insulin resistance and broader metabolic dysfunction, it may capture pathophysiological pathways relevant to AF development beyond hyperglycemia alone. In particular, lipid-related mechanism, including lipotoxicity, may promote atrial structure and electrical remodeling trough epicardial lipid accumulation, oxidative stress and low-grade inflammation, thereby facilitating AF [34,35]. This may help explain observed association between an elevated TyG index and incident AF, in contrast to the absence of an association when participants were classified solely according to glycemic status (normoglycemia, prediabetes and T2D).

Conversely, a low TyG index may result from low TG and/or low glucose levels. Inverse associations with lipid levels and AF have been demonstrated previously, but the evidence is inconsistent [36,37]. In addition, hypoglycemia has earlier been linked to increase risk for AF in adults with T2D [38] and low glucose levels could contribute to AF through mechanisms such as increased sympathetic activity, catecholamine surge and inflammation [39]. In the present study, we have tried to consider multiple interaction factors, mainly by excluding other cardiovascular comorbidities except hypertension. Besides, we tried to explore the possible interaction of obesity. Thus, while TyG-index was not found to be positively associated to incident AF, TyG-BMI index was found to have a positive association with incident AF, suggesting that the increased risk for AF could be driven from obesity rather than insulin resistance per se.

### 4.3 All-cause mortality and incident type 2 diabetes

Prediabetes and T2D were in our study associated with an increased mortality risk, as previously reported [40]. Prediabetes was also associated with an increased risk of developing T2D, with a hazard ratio of 4.5 compared to normoglycemic individuals. This is in line with data from the ARIC study, where a population, with a mean age of 75 years and prediabetes, defined as FPG 5.6–6.9 mmol/L, had approximately 3 times higher risk of T2D [41]. In addition, and consistent with earlier evidence, both elevated TyG index and elevated TyG-BMI index was positively associated with increased mortality [42,43]. Thus, although hyperglycemic status was not unequivocally associated with development of AF in our study, it remains linked to the progression of hyperglycemia and an increased risk of mortality.

### 4.4 Strengths and limitations

The strengths of our study are the large population size, long follow-up time and excellent coverage in national registers, supporting the robustness of our findings. However, there are also some important limitations. First, retrospective observational cohort studies have inherent limitations, including missing data. Of the 40 896 eligible participants in the registry, only 15 715 were included in the study, primarily due to the absence of recorded fasting glucose measurement. This reflects real-world clinical practice, in which glucose is not routinely assessed in patients with hypertension in the absence of diabetes. The paramount of missing glucose values demonstrates that the metabolic assessment in the hypertensive population is insufficient in PHC. Additionally, HbA1c data were missing for 68 % of included participants (Table S3), predominantly among those without diabetes, and HbA1c were therefore not included in our analyses. Smoking status was also excluded due to substantial missingness and inability to distinguish former from current smokers. OSA and COPD were likely substantially underdiagnosed in this cohort and therefore not included in the analyses. Furthermore, variables such as alcohol intake, physical activity and diet, all known to affect AF risk, were not available in the registry and might have resulted in residual confounding, potentially biasing the estimated associations.

In addition, many patients did not have a registered weight, height or subsequentially BMI, potentially affecting the results (Table S3). However, several sensitivity analyses to account for missing BMI did not change the main results. Second, all variables and diagnose codes were recorded as a part of routine clinical practice. Hence, measurements of variables were not formally standardized and there may be systematic differences in measurements between PHC centers, resulting in treatment bias. However, diagnose codes in the database have been validated with a sensitivity greater than 80 % for hypertension and T2D [44]. Finally, there might be surveillance bias, as we did not screen for AF using a standardized, dedicated protocol but used registered diagnose codes. This would potentially have underestimated the number of incident AF. However, patients with diabetes are more likely to have frequent regular follow-up and thereby an increased likelihood of being diagnosed with AF. These effects could not be adjusted for in this study. Future prospective studies incorporating active AF monitoring, for example through ECG wearables, may provide further insight into this knowledge gap.

## 5 Conclusion

Neither prediabetes nor T2D was independently associated with incident AF in hypertensive adults attending PHC. Moreover, the TyG index, a surrogate marker for insulin resistance, exhibit a U-shaped association with incident AF, whereas the TyG-BMI index follows a linear positive association. These findings suggest a potential role for BMI and insulin resistance in AF risk, independent of glycemic category, warranting further prospective investigation.

## Funding

The study was financed by grants from the Swedish state under the agreement between the Swedish government and the county councils, the ALF-agreements (1016427), (1010938), (965452) and (ALFGBG-1016439), and by Region Stockholm (NSV project (990472). The funding sources were not involved in the study design, research process or decision to submit for publication.

## Ethical review statement

The study was approved by the Regional Ethical Review Board in Gothenburg (approval no. 57717 and T596–18) and The Swedish Ethical Review Authority (approval no. 2019–02847 and 2021–04934).

## Data availability

The data underlying this article cannot be shared publicly due to ethical and legal restrictions from Swedish authorities. However, upon reasonable request to the authors and with permission from Swedish authorities, data can be made available to researchers who meet the criteria for access to confidential data.

## CRediT authorship contribution statement

**Sara Bentzel:** Conceptualization, Data curation, Formal analysis, Methodology, Project administration, Validation, Visualization, Writing – original draft, Writing – review & editing. **Tobias Andersson:** Conceptualization, Data curation, Formal analysis, Methodology, Resources, Writing – review & editing. **Charlotta Ljungman:** Conceptualization, Data curation, Methodology, Resources, Validation, Writing – review & editing. **Jonathan SM Johansson:** Validation, Writing – review & editing. **Per Hjerpe:** Conceptualization, Data curation, Funding acquisition, Methodology, Resources, Validation, Writing – review & editing. **Linus Schiöler:** Conceptualization, Data curation, Formal analysis, Investigation, Methodology, Software, Visualization, Writing – review & editing. **Annica Ravn-Fischer:** Conceptualization, Investigation, Methodology, Writing – review & editing. **Kristina Bengtsson Boström:** Conceptualization, Data curation, Formal analysis, Funding acquisition, Investigation, Methodology, Resources, Writing – review & editing. **Thomas Kahan:** Conceptualization, Data curation, Formal analysis, Investigation, Methodology, Resources, Validation, Writing – review & editing. **Georgios Mourtzinis:** Conceptualization, Data curation, Formal analysis, Funding acquisition, Investigation, Methodology, Resources, Supervision, Validation, Visualization, Writing – original draft.

## Declaration of competing interest

The authors declare the following financial interests/personal relationships which may be considered as potential competing interests: Thomas Kahan reports financial support was provided by Region Stockholm (NSV project (990472)). Sara Bentzel reports financial support was provided by The ALF agreements (1010938) and (965452). Sara Bentzel reports a relationship with AstraZeneca Pharmaceuticals LP that includes: funding grants and speaking and lecture fees. Sara Bentzel reports a relationship with Amarin Switzerland GmbH that includes: consulting or advisory and speaking and lecture fees. Sara Bentzel reports a relationship with Amgen Europe GmbH that includes: consulting or advisory and speaking and lecture fees. Sara Bentzel reports a relationship with Sanofi that includes: consulting or advisory and speaking and lecture fees. Sara Bentzel reports a relationship with Novo Nordisk Inc that includes: speaking and lecture fees. Sara Bentzel reports a relationship with Novartis Pharmaceuticals Corporation that includes: speaking and lecture fees. Charlotta Ljungman reports a relationship with AstraZeneca Pharmaceuticals LP that includes: speaking and lecture fees. Charlotta Ljungman reports a relationship with Pfizer Inc that includes: speaking and lecture fees. Charlotta Ljungman reports a relationship with Amgen Europe GmbH that includes: speaking and lecture fees. Charlotta Ljungman reports a relationship with Novo Nordisk Inc that includes: speaking and lecture fees. Annica Ravn-Fischer reports a relationship with Amarin Switzerland GmbH that includes: consulting or advisory and speaking and lecture fees. Annica Ravn-Fischer reports a relationship with Bayer Corporation that includes: funding grants. Annica Ravn-Fischer reports a relationship with Amgen Europe GmbH that includes: consulting or advisory and speaking and lecture fees. Annica Ravn-Fischer reports a relationship with AstraZeneca Pharmaceuticals LP that includes: consulting or advisory and speaking and lecture fees. Annica Ravn-Fischer reports a relationship with Boehringer Ingelheim Canada Ltd that includes: consulting or advisory and speaking and lecture fees. Sara Bentzel reports a relationship with Boehringer Ingelheim GmbH that includes: speaking and lecture fees. Annica Ravn-Fischer reports a relationship with Bristol Myers Squibb Co that includes: consulting or advisory and speaking and lecture fees. Annica Ravn-Fischer reports a relationship with Janssen Pharmaceuticals Inc that includes: consulting or advisory and speaking and lecture fees. Annica Ravn-Fischer reports a relationship with Eli Lilly and Company that includes: consulting or advisory and speaking and lecture fees. Annica Ravn-Fischer reports a relationship with Novartis Pharmaceuticals Corporation that includes: consulting or advisory and speaking and lecture fees. Annica Ravn-Fischer reports a relationship with Novo Nordisk Inc that includes: consulting or advisory and speaking and lecture fees. Annica Ravn-Fischer reports a relationship with Organon & Co. Inc. that includes: consulting or advisory and speaking and lecture fees. Annica Ravn-Fischer reports a relationship with Orion Pharma (UK) Ltd that includes: consulting or advisory and speaking and lecture fees. Annica Ravn-Fischer reports a relationship with Pfizer Inc that includes: consulting or advisory and speaking and lecture fees. Annica Ravn-Fischer reports a relationship with Sanofi SA that includes: consulting or advisory and speaking and lecture fees. Thomas Kahan reports a relationship with Medtronic Inc that includes: funding grants. Thomas Kahan reports a relationship with ReCor Medical Inc that includes: funding grants. If there are other authors, they declare that they have no known competing financial interests or personal relationships that could have appeared to influence the work reported in this paper.

## Appendix Supplementary materials

**Supplementary Figure S1.** Triglyceride-glucose index association to incident atrial fibrillation with imputed values for body mass index Age as the time scale, adjusted for body mass index, sex, systolic blood pressure, estimated glomerular filtration rate, concomitant cardiovascular medication and educational level. Shade areas indicate 95 % confidence interval. AF, atrial fibrillation; BMI, body mass index; TyG index, Triglyceride-glucose index. Image, application 1

**Supplementary Figure S2.** Triglyceride-glucose index association to incident atrial fibrillation stratified by different body mass index values Age as the time scale, adjusted for sex, systolic blood pressure, estimated glomerular filtration rate, concomitant cardiovascular medication and educational level. Shade areas indicate 95 % confidence interval. AF, atrial fibrillation; BMI, body mass index; TyG index, Triglyceride-glucose index. Image, application 2

Image, application 3

**Supplementary Figures S3A and S3B.** Triglyceride-glucose index association to incident atrial fibrillation in relation to sex. Age as the time scale, adjusted for body mass index, systolic blood pressure, estimated glomerular filtration rate, concomitant cardiovascular medication and educational level. Shade areas indicate 95 % confidence interval. AF, atrial fibrillation; TyG index, Triglyceride-glucose index. Image, application 4

Image, application 5
