# Supplementary figures and images for: Associations between prediabetes, type 2 diabetes and incident atrial fibrillation in patients with hypertension: Results from the Swedish Primary Care Cardiovascular Database
Source: Am J Prev Cardiol. 2026 Mar 23;28:101573. doi: 10.1016/j.ajpc.2026.101573 (PMC13325982; doi:10.1016/j.ajpc.2026.101573)

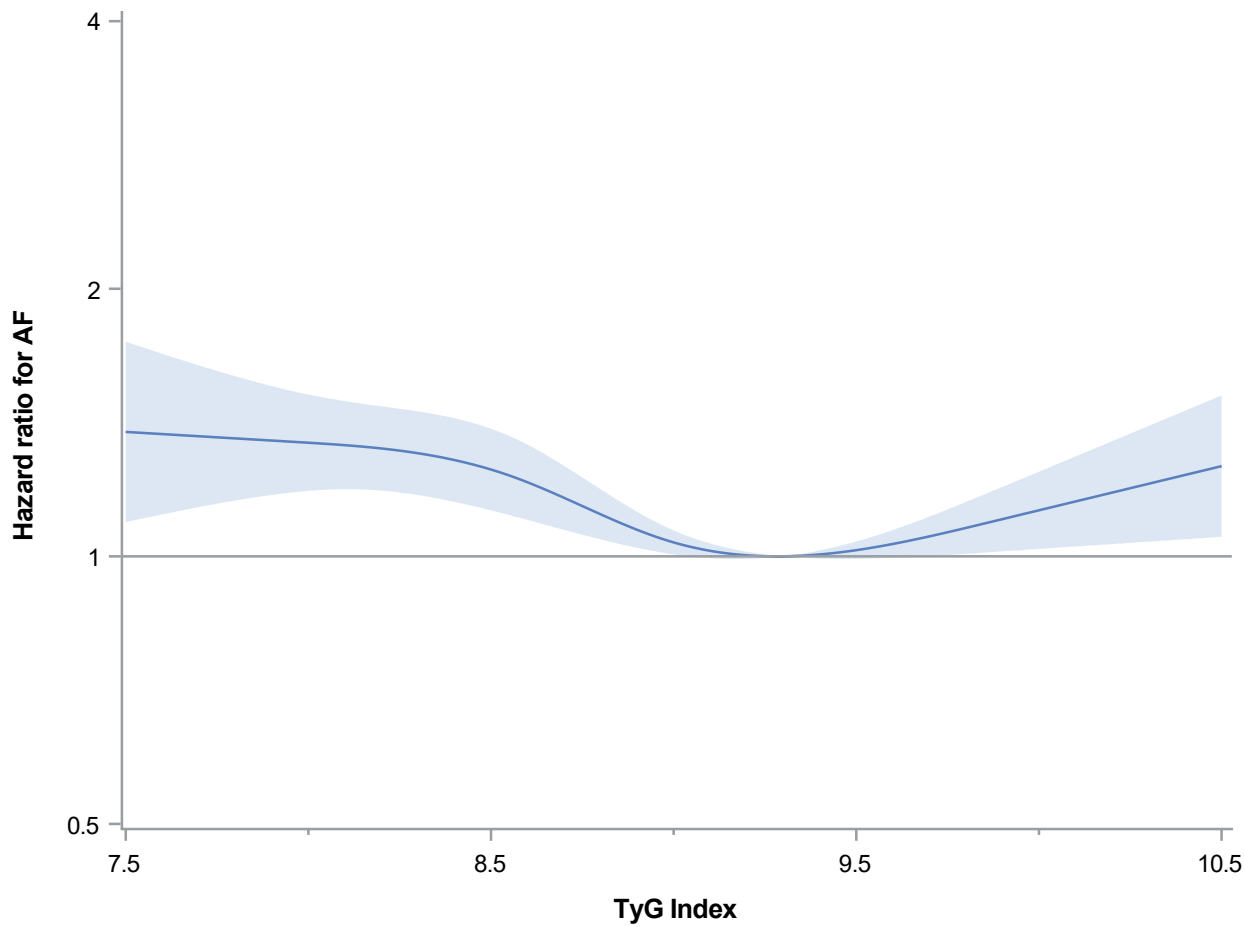

Supplement: Supplementary file 1 — Supplementary Figure S1. Triglyceride-glucose index association to incident atrial fibrillation with imputed values for body mass index Age as the time scale, adjusted for body mass index, sex, systolic blood pressure, estimated glomerular filtration rate, concomitant cardiovascular medication and educational level. Shade areas indicate 95 % confidence interval. AF, atrial fibrillation; BMI, body mass index; TyG index, Triglyceride-glucose index. [file mmc1.pdf]

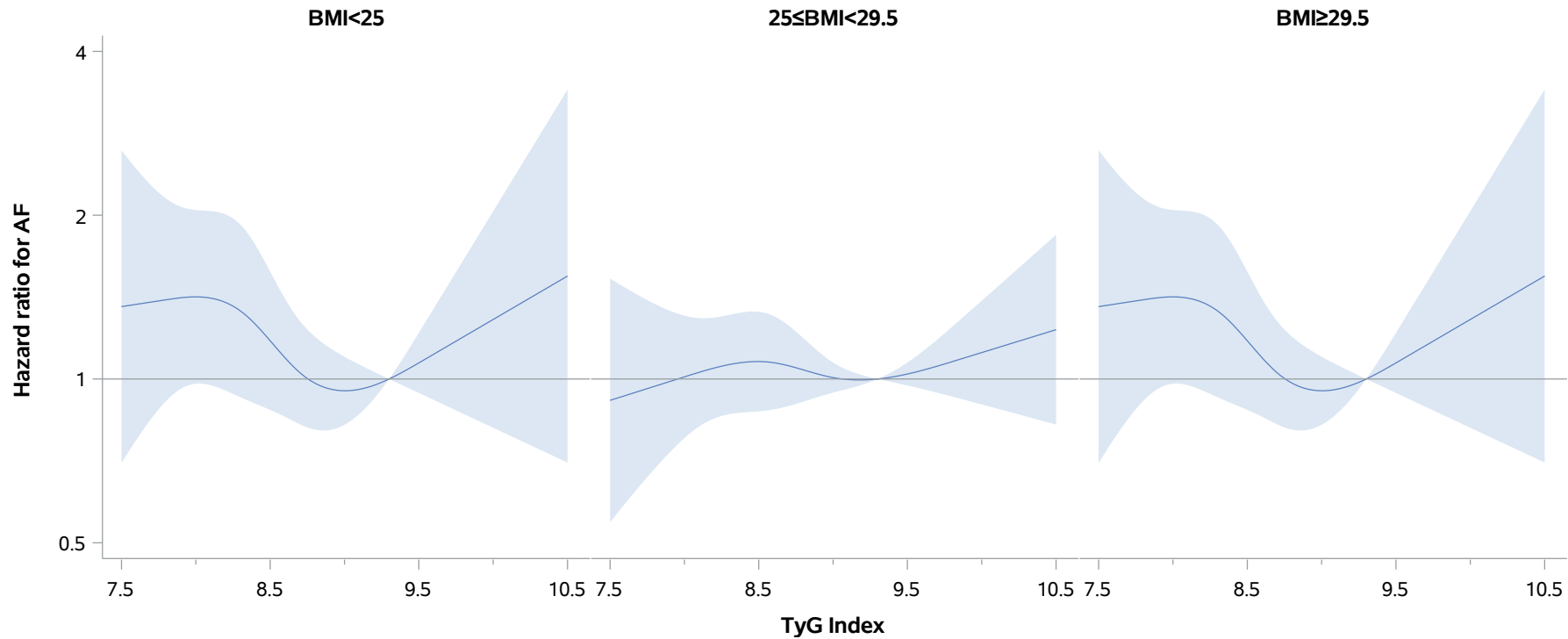

Supplement: Supplementary file 2 — Supplementary Figure S2. Triglyceride-glucose index association to incident atrial fibrillation stratified by different body mass index values Age as the time scale, adjusted for sex, systolic blood pressure, estimated glomerular filtration rate, concomitant cardiovascular medication and educational level. Shade areas indicate 95 % confidence interval. AF, atrial fibrillation; BMI, body mass index; TyG index, Triglyceride-glucose index. [file mmc2.pdf]

## Women

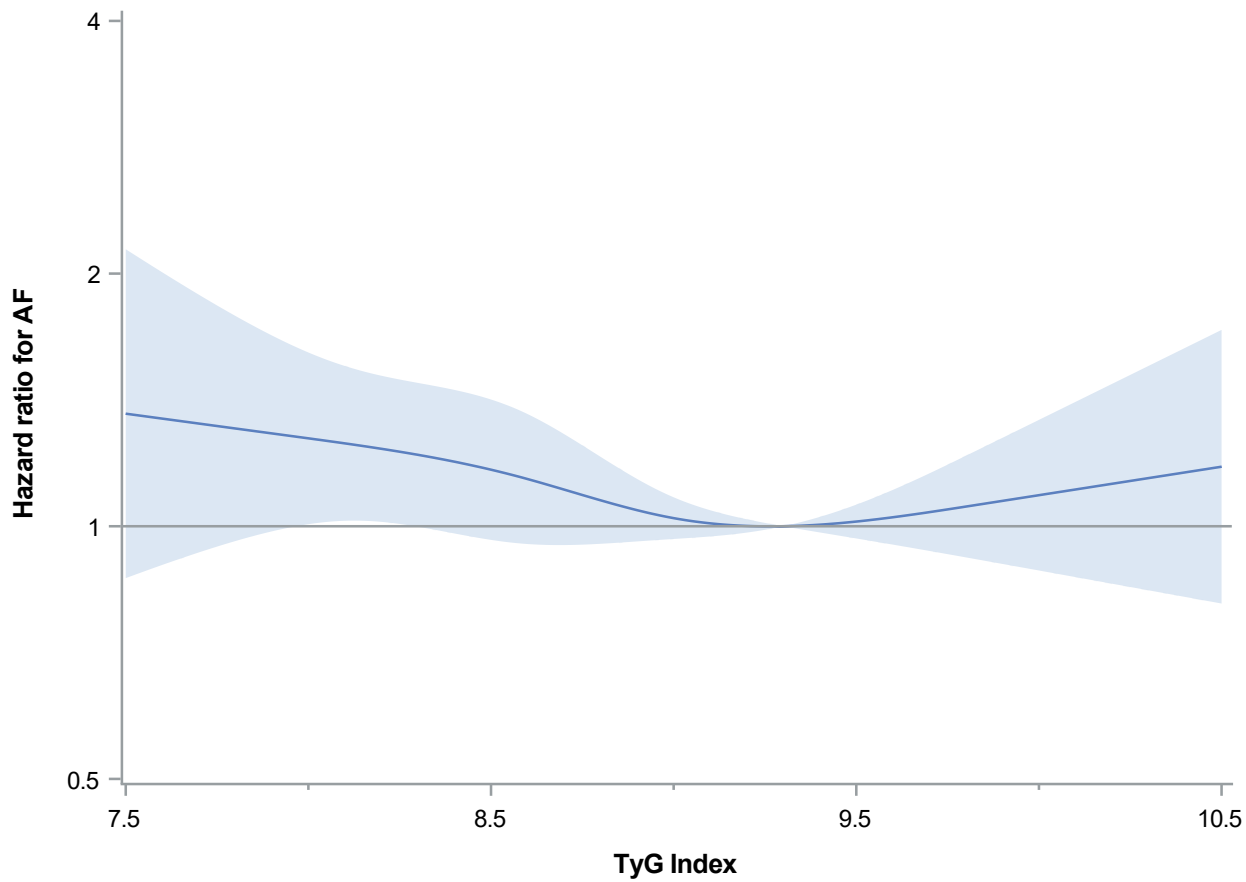

Supplement: Supplementary file 3 [file mmc3.pdf]

**Men**

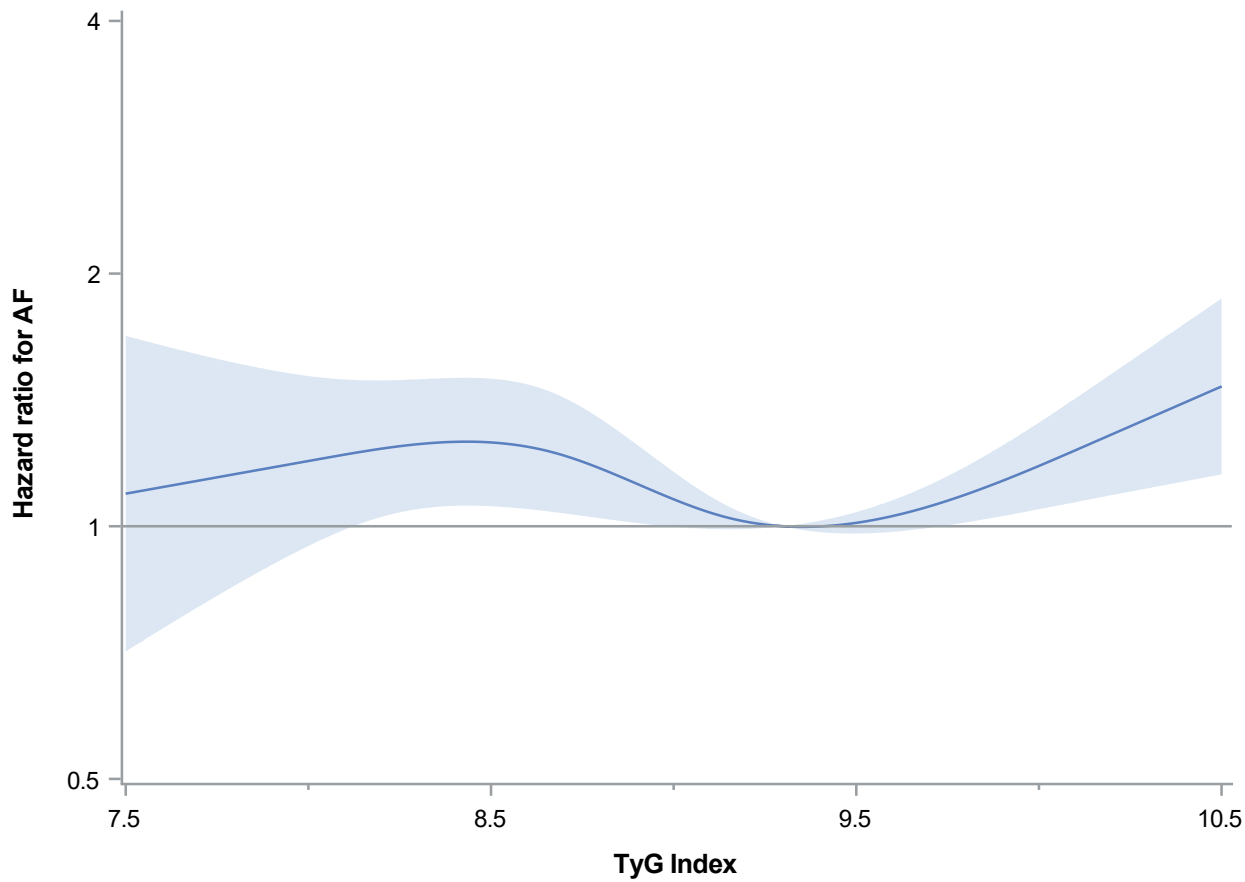

Supplement: Supplementary file 4 — Supplementary Figures S3A and S3B. Triglyceride-glucose index association to incident atrial fibrillation in relation to sex. Age as the time scale, adjusted for body mass index, systolic blood pressure, estimated glomerular filtration rate, concomitant cardiovascular medication and educational level. Shade areas indicate 95 % confidence interval. AF, atrial fibrillation; TyG index, Triglyceride-glucose index. [file mmc4.pdf]
