# Supplementary material for: Associations between prediabetes, type 2 diabetes and incident atrial fibrillation in patients with hypertension: Results from the Swedish Primary Care Cardiovascular Database
Source: Am J Prev Cardiol. 2026 Mar 23;28:101573. doi: 10.1016/j.ajpc.2026.101573 (PMC13325982; doi:10.1016/j.ajpc.2026.101573)
Supplement: Supplementary file 5 [file mmc5.docx]

**Supplementary**

| **Table S1.** Drug classes assessed and corresponding ATC codes | |
| --- | --- |
| **Drug class** | **ATC codes** |
| Angiotensin converting enzyme inhibitors  Angiotensin receptor blockers | C09A C09BA C09BB  C09C C09DA C09DB |
| Thiazide/Thiazide-like diuretics | C03A C09BA C09DA C03EA C03B |
| Loop diuretics | C03C |
| Mineralocorticoid receptor antagonists | C03D |
| Beta blockers | C07AB C07AG C07FB |
| Calcium channel blockers (any type) | C08C C09BB C09DB C07FB C08D |
| Calcium channel blockers (DHP type) | C08C C09BB C09DB C07FB |
| Calcium channel blockers (non-DHP type) | C08D |
| Other anti-hypertensive drugs | C02CA04 C02AC05 |
| Statins | C10AA C10BA |
| Ezetimibe | C10AX09 C10BA02 C10BA05 C10BA06 |
| Acetylsalicylic acid | B01AC06 |
| P2Y12 inhibitors | B01AC04 B01AC22 B01AC24 |
| Anticoagulants | B01AA03 B01AE07 B01AF |
| ATC, Anatomical Therapeutic Chemical classification system; DHP, dihydropyridine | |

| **Table S2.** Previous studies and results on the association between prediabetes/diabetes and risk of atrial fibrillation | | | | | | |
| --- | --- | --- | --- | --- | --- | --- |
| **Association reported** | **Author/Journal/Doi** | **Study/design** | **Population** | **Exposure definition** | **Key finding (direction)** | **Comment on comparability** |
| **Positive** | Li et al/Frontiers in Endocrinology/ 10.3389/fendo.2025.1763810 | Systematic review & meta-analysis (2025) | >15 million participants; cohort studies | Prediabetes vs normoglycemia (various definitions) | Prediabetes associated with higher AF risk (pooled HR ~1.20) | Substantial heterogeneity across definitions and populations |
| **Positive** | Lind et al/ Cardiovascular Diabetology/10.1186/s12933-021-01422-3 | AMORIS cohort (Sweden), prospective (2021) | General population health examinations; no prior CVD | IFG/dysglycemia by fasting glucose | Increase glucose value associated with incident AF | Association partly attenuated by BMI/comorbidities in some models (as discussed in paper) |
| **Positive** | Kim et al/Heart/10.1136/heartjnl-2022-322094 | Two-cohort analysis (K-NHIS HealS + UK Biobank) (2023) | Large screening cohorts (176,937 & 167,946 participants) | IFG + BP categories | IFG associated with increased AF risk (dose–response) | Different setting than a fully hypertensive cohort |
| **Positive** | Aune et al/Journal of Diabetes and Its Complications/10.1016/j.jdiacomp.2018.02.004 | Systematic review & meta-analysis (2018) | Multiple observational cohorts | Diabetes and/or glucose measures | Overall diabetes/hyperglycemia associated with higher AF risk | Includes heterogeneous designs/adjustment; not specific to hypertensive-only samples |
| **Positive** | Ahmadi et al/Cardiovasc Diabetol/10.1186/s12933-019-0983-1 | Nationwide cohort (Sweden) (2019) | T2D vs matched controls | T2D; stratified by glycemic control/renal disease | T2D associated with higher AF risk (HR 1.28); risk varies by control/complications | Not directly comparable to studies using normoglycemia/prediabetes/T2D within one cohort. No adjustment for BMI |
| **Null / attenuated** | Johansson et al/Journal of Internal Medicine/10.1111/joim.13688 | Nationwide cohort (Sweden) (2023) | Large screening cohort (88,889 participants) | Normoglycemia vs IFG/IGT/Unknown DM2/DM2 | No association after full adjustment | Predominately participants without hypertension |
| **Null / attenuated** | Latini et al/American Heart Journal/ 10.1016/j.ahj.2013.08.012 | NAVIGATOR post-hoc analysis (2013) | Trial population with impaired glucose tolerance | Glucose measures and progression to diabetes | Progression to diabetes not associated with incident AF after accounting for other factors | Selected high-risk trial population; different ascertainment and covariate structure |
| **Null (current study)** |  | Present study | Fully hypertensive population | Normoglycemia vs prediabetes vs T2D | Prediabetes/T2D not associated with incident AF in fully adjusted models | May reflect categorical exposure definitions and confounding control |

Studies differ in AF ascertainment (registries/ECG/clinical), glycemic definitions (IFG/IGT/HbA1c), and covariate adjustment (notably BMI and comorbidities), likely contributing to heterogeneity and apparent discrepancies.

| **Table S3.** Missing values according to glycemic status | | | | |
| --- | --- | --- | --- | --- |
|  | All  (n=15 715) | Normal glycemic status (n=9353) | Prediabetes  (n=2689) | Diabetes mellitus type 2  (n=3673) |
| HbA1c | 10 630 (68) | 8302 (89) | 785 (71) | 424 (12) |
| BMI | 6117 (39) | 4300 (46) | 1192 (44) | 625 (17) |
| Smoking | 2158 (14) | 1611 (17) | 409 (15) | 138 (4) |
| eGFR | 604 (4) | 389 (4) | 108 (4) | 107 (3) |
| Total cholesterol | 1459 (9) | 755 (8) | 298 (11) | 406 (11) |
| Triglyceride | 1634 (10) | 857 (9) | 329 (12) | 448 (12) |
| TyG index | 1634 (10) | 857 (9) | 329 (12) | 448 (12) |
| TyG-BMI index | 6830 (43) | 4601 (49) | 1303 (48) | 926 (25) |
| Values are presented as numbers (%) of participants with missing data | | | | |
